# Association between food insecurity and Parkinson’s disease: A cross-sectional analysis of nationally representative data

**DOI:** 10.1097/MD.0000000000048488

**Published:** 2026-04-24

**Authors:** Rui He, Linling Lu, Jinying Wang

**Affiliations:** a Department of Neurology, Shaoxing Second Hospital, Shaoxing, Zhejiang, China; b Department of Internal Medicine, Shaoxing Second Hospital, Shaoxing, Zhejiang, China.

**Keywords:** food insecurity, NHANES, Parkinson’s disease, public health

## Abstract

Parkinson’s disease (PD) is a prevalent neurodegenerative disorder that imposes a substantial burden on patients and healthcare systems worldwide. Food insecurity, defined as limited or uncertain access to adequate and nutritious food, has been associated with multiple chronic health conditions; however, its relationship with PD has not been well explored. This study aimed to investigate the association between food insecurity and PD using nationally representative data from the National Health and Nutrition Examination Survey (NHANES) 2007 to 2018. A total of 25,714 participants were included after excluding individuals with incomplete information. Household food insecurity was assessed using the U.S. Food Security Survey Module and categorized into four levels: full, marginal, low, and very low food security. Weighted logistic regression models were applied to evaluate the association between food insecurity and PD while adjusting for demographic, socioeconomic, lifestyle, and health-related covariates. The results showed that food insecurity was significantly associated with higher odds of PD (adjusted OR 1.85; 95% CI 1.26–2.74; *P* = .0002). In the four-category analysis, compared with full food security, low food security (OR 2.01; 95% CI 1.24–3.25; *P* = .005) and very low food security (OR 2.13; 95% CI 1.23–3.68; *P* = .01) were associated with increased odds of PD, whereas marginal food security was not statistically significant. These findings suggest that severe food insecurity may represent a modifiable risk factor for Parkinson’s disease, highlighting the importance of public health interventions aimed at improving food access and reducing the burden of PD.

## 1. Introduction

Parkinson’s disease (PD) is a prevalent neurodegenerative disorder and a leading chronic condition globally. It is characterized by progressive motor symptoms due to decreased dopamine levels, resulting from the loss of dopaminergic neurons in the substantia nigra.^[1]^

The Global Burden of Disease (GBD) study reported 1.02 million cases of PD in 2017.^[2]^ Additionally, the report indicated that in 2016, 6.1 million people were living with PD worldwide, with the age-standardized prevalence rate (ASR) increasing by 21.7% from 1990 to 2016.^[3]^ Research predicts that the burden of PD will rise significantly in the coming decades.^[4]^ For instance, it is estimated that by 2030, 4.94 million people in China will be living with PD, accounting for half of the global patient population.^[5]^ The rapid progression of PD has imposed a substantial burden on society, individuals, and healthcare systems.^[6–8]^

Food insecurity is defined as a lack of social, economic, and physical access to adequate and nutritious food necessary to sustain an active and healthy lifestyle.^[9,10]^ Food security is widely recognized as a fundamental determinant of health, because consistent access to sufficient and nutritious food is essential for maintaining normal metabolic function, immune regulation, and psychological well-being. In contrast, food insecurity is considered a major public health concern, as it may contribute to poor diet quality, micronutrient deficiencies, chronic stress, and increased vulnerability to a broad range of chronic diseases. In recent years, food insecurity has been closely linked to multiple aspects of physical health.^[11–14]^ A study of 3632 participants suggested that individuals facing food insecurity may be more likely to suffer from sarcopenia compared to those with adequate food security.^[15]^ A large cross-sectional study by Xiao, using data from the National Health and Nutrition Examination Survey (NHANES), demonstrated a strong positive correlation between food insecurity and the incidence of overactive bladder syndrome.^[9]^ Numerous studies have documented a significant association between food insecurity and conditions such as obesity and cardiovascular disease.^[13,16]^

Beyond physical health, food insecurity can also impact mental health.^[17]^ Individuals suffering from food insecurity may experience feelings of deprivation, anxiety about food availability and restricted food choices.^[18]^ Montgomery et al.‘s cross-sectional study based on NHANES data highlighted a significant association between food insecurity and depressive symptoms in patients with diabetes and prediabetes.^[19]^ A similar study representative of Korea also found significant links between food insecurity, poor mental health indicators, and declining basic living conditions.^[20]^

In summary, food insecurity appears to affect a wide range of physical and psychological issues. Evidence suggests that food insecurity can lead to dysbiosis in gut flora, increased intestinal permeability, and heightened neuroinflammation, which may influence the progression and pathogenesis of PD.^[21,22]^ However, recent studies have not explored the relationship between food insecurity and PD. Consequently, a cross-sectional analysis using extensive NHANES data to investigate this relationship is warranted. Our research hypothesis proposes a positive correlation between food insecurity and PD.

## 2. Methods

### 2.1. Study population

This cross-sectional study utilized data from the NHANES database for the 2007–2018 cycle, which includes comprehensive sociodemographic data, health behaviors, and medical histories of participants in the United States. NHANES employs a stratified, random, multistage, and probabilistic clustering design to select households for interviews. Physical examinations and laboratory sample collections, including blood analyses, were conducted at mobile examination centers (MECs). Ethical approval for NHANES was granted by the National Center for Health Statistics (NCHS) Ethics Review Board, and all participants provided written informed consent prior to participation. The present study was a secondary analysis of publicly available, de-identified NHANES data; therefore, no additional institutional review board approval was required.

### 2.2. Exclusion criteria and final sample

**Figure 1.**
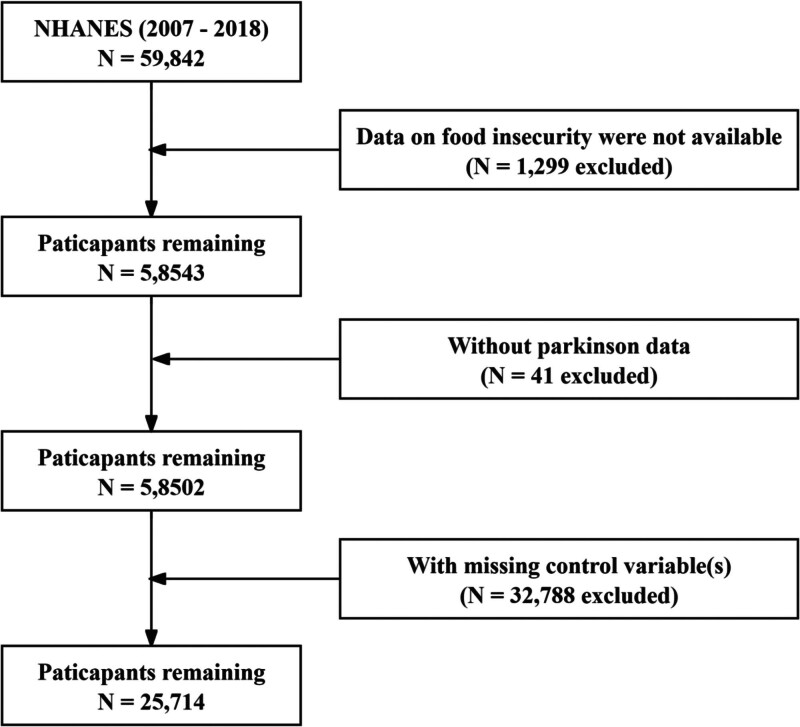
The selection process of NHANES 2007–2018.

The initial NHANES 2007–2018 dataset comprised 59,842 participants. After excluding individuals without valid food insecurity data (n = 1299), those lacking Parkinson’s disease data (n = 41), and participants with missing control variables (n = 32,788), the final analytic sample consisted of 25,714 participants. A flowchart detailing the study population screening is depicted in Figure 1.

### 2.3. Explanatory variables

Household food security in the United States was assessed using the US Food Security Survey Module (FSSM), which is considered the standard for measuring food security. Food security refers to consistent access to sufficient, safe, and nutritious food necessary for an active and healthy life, whereas food insecurity represents limited or uncertain availability of nutritionally adequate food. The FSSM consists of 10 questions for households with and without children, as shown in Supplementary Table 1, Supplemental Digital Content, https://links.lww.com/MD/R786. Food security status was determined based on the number of affirmative responses in the adult module: (I) Full food security was indicated by zero affirmative responses. (II) Marginal food security was indicated by 1–2 affirmative responses. (III) Low food security was indicated by 3–5 affirmative responses. (IV) Very low food security was indicated by 6–10 affirmative responses. Following USDA guidelines, food security was categorized as a binary variable: food security (full and marginal food security) and food insecurity (low and very low food security).

### 2.4. Outcome variable

The outcome variable for this study was Parkinson’s disease (PD). In the NHANES database, PD cases were identified based on self-reported anti-PD medications.^[23,24]^ PD was defined if individuals reported using any of the following PD drugs: Benztropine, Methyldopa, Carbidopa, Levodopa, Entacapone, Amantadine, and Ropinirole.

### 2.5. Covariates

Demographic variables (age, sex, race), socioeconomic variables (marital status, educational level, poverty income ratio [PIR]), and health-related variables (body mass index [BMI], drinking status, smoking status, chronic medical diseases, and recreational activity) were included as covariates. Chronic medical conditions encompassed hypertension, diabetes, and cardiovascular disease (CVD).

### 2.6. Statistical analysis

All statistical analyses were conducted using R software (version 4.2.3), adhering to NHANES guidelines by applying appropriate sample weights to adjust for the complex, multistage sampling design. Weighted means with standard errors (SE) were reported for continuous variables, while weighted percentages (%) were used for categorical variables. Differences between food security groups were assessed using weighted t-tests for continuous variables and weighted chi-squared tests for categorical variables. The association between food security and PD was examined through weighted univariate and multivariate logistic regression analyses. Four weighted logistic regression models were developed: Model 1: Unadjusted. Model 2: Adjusted for age, sex, and race. Model 3: Further adjusted for marital status, education level, BMI, recreational activity, smoking status, and drinking status. Model 4: Additional adjustment for hypertension, diabetes, and CVD. Subgroup analyses were conducted across various demographic and health-related variables, with interaction analysis performed to assess differential associations by including interaction terms in regression models. Odds ratios (ORs) and 95% confidence intervals (CIs) were calculated, with *P* < .05 considered statistically significant.

## 3. Results

### 3.1. Characteristics of the study population

Our study encompassed a total of 25,714 participants, among whom 259 were diagnosed with Parkinson’s disease (PD), while 25,455 were not. The study population was evenly split between males and females, with a median age of 47.26 years. The racial composition was predominantly White (68.34%), followed by Black (10.63%), Mexican (8.07%), and other races (12.97%). Detailed characteristics of the study population are presented in Table 1.

**Table 1:** Sociodemographic characteristics of US adults by food security status, NHANES 2007–2018.

Characteristic	Food security group					
	Total	Full security	Marginal security	Low security	Very low security	*P - value*
Total patients, n (%)	25,714	17,587 (68.39)	3135 (12.19)	3188 (12.40)	1788 (6.95)	
Age, years, Mean (SD)	47.26 ± 0.28	48.85 ± 0.30	42.60 ± 0.52	42.40 ± 0.52	41.59 ± 0.59	< 0.0001
Sex, n (%)						0.002
Male	12782 (49.14)	8888 (49.82)	1492 (46.34)	1509 (46.12)	893 (49.74)	
Female	12932 (50.86)	8699 (50.18)	1659 (53.66)	1679 (53.88)	895 (50.26)	
Race, n (%)						< 0.0001
White	11144 (68.34)	8545 (74.26)	948 (49.04)	963 (47.61)	688 (54.87)	
Black	5381 (10.63)	3340 (8.55)	803 (17.09)	769 (16.76)	469 (17.75)	
Mexiacan	3717 (8.07)	2005 (5.70)	695 (15.44)	708 (17.38)	309 (12.44)	
Other	5472 (12.97)	3697 (11.49)	705 (18.43)	748 (18.26)	322 (14.95)	
Marital status, n (%)						< 0.0001
married/living with partner	15273 (63.32)	11048 (66.83)	1747 (55.11)	1651 (52.51)	827 (47.21)	
single/divorced/widowed	10441 (36.68)	6539 (33.17)	1404 (44.89)	1537 (47.49)	961 (52.79)	
Education level, n (%)						< 0.0001
less than high school	5869 (14.06)	3040 (10.15)	954 (22.01)	1237 (30.44)	638 (27.05)	
high school or equivalent	5864 (23.13)	3737 (21.06)	842 (30.82)	819 (28.25)	466 (29.74)	
college or above	13981 (62.81)	10810 (68.79)	1355 (47.17)	1132 (41.31)	684 (43.20)	
BMI, n (%)						< 0.0001
under/normal weight (< 25.00)	7312 (29.03)	5232 (29.74)	806 (26.36)	790 (25.14)	484 (30.36)	
overweight (25–29.99)	8410 (32.44)	5930 (33.66)	1024 (30.32)	942 (28.62)	514 (25.62)	
obese (≥ 30.00)	9992 (38.53)	6425 (36.61)	1321 (43.31)	1456 (46.24)	790 (44.02)	
PIR, n (%)						< 0.0001
≤ 1.00	5490 (14.02)	2197 (7.59)	1030 (26.94)	1371 (36.25)	892 (43.24)	
1.01–3.99	13581 (48.27)	9098 (44.77)	1904 (63.39)	1707 (57.95)	872 (53.91)	
≥ 4.00	6643 (37.71)	6292 (47.64)	217 (9.67)	110 (5.80)	24 (2.85)	
Smoking status, n (%)						< 0.0001
never	14130 (55.78)	10147 (58.42)	1666 (51.86)	1619 (49.62)	698 (36.41)	
former	6236 (24.76)	4629 (26.50)	660 (20.34)	625 (19.39)	322 (17.40)	
now	5348 (19.46)	2811 (15.08)	825 (27.80)	944 (30.99)	768 (46.19)	
Drinking status, n (%)						< 0.0001
never	3567 (10.20)	2315 (9.62)	499 (11.91)	535 (13.57)	218 (9.64)	
former	4025 (11.56)	2684 (11.12)	501 (12.07)	508 (13.00)	332 (14.49)	
now	18122 (78.24)	12588 (79.27)	2151 (76.01)	2145 (73.43)	1238 (75.87)	
Recreational activity, n (%)						< 0.0001
inactivity	13191 (44.73)	8358 (41.10)	1813 (52.65)	1912 (58.92)	1108 (57.37)	
moderate	6733 (28.13)	4977 (30.01)	688 (22.69)	717 (22.09)	351 (21.70)	
vigorous	5790 (27.15)	4252 (28.89)	650 (24.66)	559 (18.99)	329 (20.93)	
Hypertension, n (%)						0.04
no	14690 (62.11)	9979 (61.81)	1881 (65.50)	1831 (61.56)	999 (61.14)	
yes	11024 (37.89)	7608 (38.19)	1270 (34.50)	1357 (38.44)	789 (38.86)	
Diabetes, n (%)						0.003
no	20834 (85.67)	14370 (86.18)	2528 (84.58)	2511 (82.90)	1425 (85.02)	
yes	4880 (14.33)	3217 (13.82)	623 (15.42)	677 (17.10)	363 (14.98)	
CVD, n (%)						< 0.001
no	22958 (91.66)	15764 (92.06)	2840 (92.13)	2808 (89.43)	1546 (88.98)	
yes	2756 (8.34)	1823 (7.94)	311 (7.87)	380 (10.57)	242 (11.02)	
Parkinson, n(%)						0.01
no	25455 (99.06)	17419 (99.18)	3122 (98.97)	3154 (98.58)	1760 (98.49)	
yes	259 (0.94)	168 (0.82)	29 (1.03)	34 (1.42)	28 (1.51)	

BMI = body mass index, CVD = cardiovascular disease, PIR = poverty income ratio.

### 3.2. Comparison of participants within different food security groups

Significant differences were observed in several demographic and health-related variables across different food security groups. These variables included age (*P* < .0001), sex (*P* = .002), race (*P* < .0001), marital status (*P* < .0001), education level (*P* < .0001), BMI (*P* < .0001), PIR (*P* < .0001), smoking status (*P* < .0001), drinking status (*P* < .0001), recreational activity (*P* < .0001), hypertension (*P* = .04), diabetes (*P* = .003), cardiovascular disease (*P* < .0001), and PD (*P* = .01). These findings are summarized in Table 1.

### 3.3. Association between food insecurity and Parkinson’s disease

The results of the multilevel logistic regression analysis examining the association between food security and PD are displayed in Table 2. When food security was classified as a dichotomous variable (food security and food insecurity), we found that food insecurity significantly increased the risk of PD (OR, 1.85; 95% CI, 1.26–2.74; *P* = .0002), after adjusting for various confounders including age, sex, race, marital status, education level, BMI, recreational activity, smoking status, drinking status, hypertension, diabetes, and cardiovascular disease.

**Table 2:** Logistic regression analysis of food security and Parkinson.

	Model 1	*P*-value	Model 2	*P*-value	Model 3	*P*-value	Model 4	*P*-value
	OR (95%CI)		OR (95%CI)		OR (95%CI)		OR (95%CI)	
Food security								
Food security	Reference		Reference		Reference		Reference	
Food insecurity	1.72 (1.18,2.53)	0.01	2.53 (1.72,3.72)	<0.0001	1.92 (1.30,2.85)	0.001	1.85 (1.26,2.74)	0.0002
Classification of food security								
Full security	Reference		Reference		Reference		Reference	
Marginal security	1.26 (0.72,2.21)	0.42	1.89 (1.05,3.42)	0.03	1.57 (0.85,2.90)	0.15	1.55 (0.84,2.87)	0.16
Low security	1.73 (1.05,2.86)	0.02	2.67 (1.62,4.39)	<0.001	2.11 (1.29,3.46)	0.004	2.01 (1.24,3.25)	0.005
Very low security	1.85 (1.11,3.07)	0.01	2.90 (1.74,4.84)	<0.0001	2.23 (1.30,3.83)	0.004	2.13 (1.23,3.68)	0.01
P for trend		0.002		<0.0001		<0.0001		<0.001

Model1: unadjusted.

Model2: adjusted for age, sex, and race.

Model3: adjusted for age, sex, race, marital status, education level, BMI, recreational activity, smoking status, and drinking status.

Model4: further adjusted for hypertension, diabete, and CVD.

CI = confidence intervals, OR = odds ratios.

When food security was divided into four categories, compared with the full security group, the risk of PD was 1.55 (95% CI, 0.84–2.87; *P* = .16), 2.01 (95% CI, 1.24–3.25; *P* = .005), and 2.13 (95% CI, 1.23–3.68; *P* = .01) times higher in the marginal security, low security, and very low security groups, respectively, after adjusting for the same confounders.

Subgroup analyses revealed no significant interaction between PD and other covariates in relation to depression, as illustrated in Table 3.

**Table 3:** Association between food security and parkinson in different subgroups.

Variables	Adjusted Model 4		
	OR (95 CI%)		P for interaction
	Food security	Food insecurity	
Age			0.02
20–39	Reference	3.01 (1.06, 8.55)	
40–59	Reference	2.25 (1.28,3.94)	
≥ 60	Reference	0.73 (0.27,1.97)	
Sex			0.11
Male	Reference	1.19 (0.63,2.25)	
Female	Reference	2.23 (1.40,3.54)	
Race			0.57
White	Reference	2.21 (1.38,3.52)	
Black	Reference	0.91 (0.44, 1.89)	
Mexiacan	Reference	2.72 (1.21, 6.10)	
Other	Reference	1.06 (0.41, 2.71)	
Marital status			0.98
married/living with partner	Reference	1.96 (1.08, 3.55)	
single/divorced/widowed	Reference	1.79 (1.11,2.90)	
Education level			0.05
less than high school	Reference	2.16 (1.33,3.49)	
high school or equivalent	Reference	0.94 (0.41,2.14)	
college or above	Reference	2.47 (1.35,4.51)	
BMI			0.26
under/normal weight (< 25.00)	Reference	1.95 (0.74,5.13)	
overweight (25–29.99)	Reference	3.26 (1.65,6.42)	
obese (≥ 30.00)	Reference	1.42 (0.84,2.39)	
PIR			0.43
≤ 1.00	Reference	1.47 (0.85,2.52)	
1.01–3.99	Reference	2.00 (0.95, 4.21)	
≥ 4.00	Reference	3.56 (0.98,12.96)	
Smoking status			0.49
never	Reference	1.62 (0.83,3.16)	
former	Reference	1.30 (0.53,3.20)	
now	Reference	2.12 (1.03, 4.39)	
Drinking status			0.64
never	Reference	1.65 (0.52,5.26)	
former	Reference	1.76 (0.99,3.15)	
now	Reference	1.61 (0.98,2.62)	
Recreational activity			0.61
inactivity	Reference	1.61 (1.08,2.39)	
moderate	Reference	4.09 (1.22,13.65)	
vigorous	Reference	1.38 (0.58, 3.27)	
Hypertension			0.31
no	Reference	2.40 (1.22,4.74)	
yes	Reference	1.31 (0.84,2.03)	
Diabetes			0.94
no	Reference	1.77 (1.06,2.98)	
yes	Reference	1.47 (0.81, 2.65)	
CVD			0.63
no	Reference	1.82 (1.22,2.72)	
yes	Reference	1.21 (0.63,2.34)	

BMI = body mass index, PIR, poverty income ratio, CI = confidence intervals, CVD = cardiovascular disease, OR = odds ratios. Analyses were adjusted for age, sex, race, marital status, education level, BMI, PIR, recreational activity, smoking status, drinking status, hypertension, diabete, and CVD.

## 4. Discussion

This study is the first large-scale cross-sectional analysis to explore the relationship between food insecurity and Parkinson’s disease (PD). Our findings indicate that food insecurity, particularly low and very low food security, is significantly associated with an increased likelihood of developing PD. These associations remained significant even after adjusting for demographics, socioeconomic factors, BMI, drinking status, smoking status, chronic diseases, and potential covariates related to recreational activities. Our study identifies food insecurity as a new modifiable risk factor for the development of Parkinson’s disease.

Our results showed a significant association between food insecurity and PD (OR, 1.85; 95% CI, 1.26–2.74; *P* = .0002) after adjusting for all potential confounders. Furthermore, a sensitivity analysis categorizing food insecurity into four groups revealed that participants with very low food security had a 101% higher risk of PD compared to those with full food security. Additionally, individuals with very low food security had a 113% greater risk of experiencing PD. Currently, the link between food insecurity and PD may be related to several mechanisms.

Food insecurity is increasingly recognized as an important social determinant of health, with broad impacts that extend beyond nutritional deprivation. Limited or uncertain access to adequate food may contribute to poor diet quality, micronutrient deficiencies, chronic psychological stress, and metabolic disturbances. Previous studies have linked food insecurity to a wide range of adverse health outcomes, including obesity, diabetes, cardiovascular disease, and mental health disorders. These systemic effects suggest that food insecurity may create long-term physiological vulnerability, which could increase susceptibility to neurodegenerative conditions such as Parkinson’s disease.

In recent years, the potential impact of diet on PD risk has garnered public attention.^[25,26]^ A study by Liu et al found that higher dietary iron intake was linked to a greater risk of PD, whereas higher vitamin intake had the opposite effect.^[25]^ Similarly, research by Nam et al suggested that components of metabolic syndrome, such as hypertriglyceridemia, low high-density lipoprotein cholesterol, and hyperglycemia, were positively associated with PD risk.^[27]^ A prospective observational cohort study by Mischley et al involving 1053 subjects found that fresh vegetables, fresh fruits, nuts and seeds, non-fried fish, olive oil, wine, coconut oil, fresh herbs, spices, coenzyme Q10, and fish oil reduced the progression of PD, whereas canned fruits and vegetables, dietary soda, fried foods, beef, ice cream, yogurt, cheese, and iron were associated with more rapid PD progression.^[28]^ Another prospective population-based cohort study among 5289 subjects reported that intakes of total fat, monounsaturated fatty acids, and polyunsaturated fatty acids were significantly associated with a lower risk of PD.^[29]^ Food insecurity is mainly characterized by the consumption of high-fat fast foods, high levels of sugar, and reduced intake of vegetables and fruits. This high-calorie, low-nutrient diet leads to inadequate nutrient intake and an elevated risk of various forms of malnutrition, thereby increasing the incidence of PD.^[30]^

There is also growing interest in predicting disease risk through dietary patterns. A recent Swedish cohort study consistently associated a higher Mediterranean diet score with a lower risk of PD.^[31]^ Adopting high-quality dietary patterns, such as the Mediterranean-DASH Diet Intervention for Neurodegenerative Delay (MIND), is associated with a reduced risk of cognitive decline.^[32]^ Increased intake of fruits and vegetables may improve cognitive function due to their antioxidant properties. Dietary intake profoundly influences the diversity and functionality of the microbiota within the human gastrointestinal tract.^[33,34]^ Typical features of food insecurity, such as elevated sugar intake and reduced consumption of complex carbohydrates and fiber, can disrupt the homeostasis and maintenance of gut microbiota.^[35]^ Dysregulation of gut microbiota structure and composition can lead to increased intestinal permeability, amplified neuroinflammation, and elevated oxidative stress. These physiological changes are significant factors in the onset and progression of neurodegenerative diseases, including Parkinson’s disease.^[36,37]^

In addition to its nutritional implications, food insecurity can predispose individuals to anxiety and depression due to the stress and uncertainty about their next meal.^[17]^ Several studies have identified a significant positive link between food insecurity and depression in both cross-sectional and longitudinal analyses. A study by Leentjens et al found that patients diagnosed with depression had a higher probability of subsequently being diagnosed with PD compared to a control population.^[38,39]^ Depression is also considered one of the most common comorbidities in patients with PD. The stress associated with food insecurity can affect overall health, creating a vicious cycle where poor physical and mental health exacerbate economic and physical hardship. Thus, addressing food insecurity requires a comprehensive approach that includes reducing income inequality and implementing robust food programs and services, which may help prevent PD.

The study has several strengths and limitations. A significant strength is the large sample size, which enhances the generalizability of the findings to the national population. Additionally, the use of appropriate sampling weights in our analysis helps mitigate biases associated with oversampling, thereby strengthening the reliability of our conclusions. However, the study also has limitations. Firstly, the data on food insecurity and PD were self-reported by participants, as constrained by the NHANES database, potentially introducing information bias. Secondly, the cross-sectional design of the study prevents establishing causality between food insecurity and PD. Lastly, there remains a possibility of unmeasured confounding factors that were not accounted for in the study.

## 5. Conclusions

In this study, we identified a positive association between food insecurity and the incidence of PD. Moreover, we found that food insecurity, especially at more severe levels, substantially increases the likelihood of developing PD. Previous research has linked food insecurity to various chronic conditions such as obesity, hyperlipidemia, and hypertension. This research is the first to establish a direct link between food insecurity and PD. Addressing food insecurity could be a crucial step in reducing the incidence of PD and improving overall health outcomes. Policymakers should prioritize initiatives that enhance food accessibility and quality. By integrating food security considerations into public health policies, we can potentially mitigate the risk of Parkinson’s disease and address a significant public health challenge.

## Author contributions

**Conceptualization:** Rui He, Linling Lu, Jinying Wang.

**Data curation:** Rui He, Linling Lu.

**Formal analysis:** Rui He, Linling Lu.

**Writing – original draft:** Rui He, Jinying Wang.

**Writing – review & editing:** Linling Lu, Jinying Wang.

**Methodology:** Jinying Wang.

**Resources:** Jinying Wang.

## Supplementary Material
