# Supplementary material for: Association between food insecurity and Parkinson’s disease: A cross-sectional analysis of nationally representative data
Source: Medicine (Baltimore). 2026 Apr 24;105(17):e48488. doi: 10.1097/MD.0000000000048488 (PMC13124339; doi:10.1097/MD.0000000000048488)
Supplement: Supplementary file 1 [file medi-105-e48488-s001.pdf]

Supplementary Table 1. Questions assessing the adult food security in the U.S. Food Security Survey Module.

**Questions about food conditions of the household as a whole**
1. “We worried whether our food would run out before we got money to buy more.” Was that often, sometimes, or never true for you in the last 12 months?
2. “The food that we bought just didn’t last and we didn’t have money to get more.” Was that often, sometimes, or never true for you in the last 12 months?
3. “We couldn’t afford to eat balanced meals.” Was that often, sometimes, or never true for you in the last 12 months?
**Questions about food conditions of adults in the household**
4. In the last 12 months, did you or other adults in the household ever cut the size of your meals or skip meals because there wasn’t enough money for food? (Yes/No)
5. (If yes to question 4) How often did this happen—almost every month, some months but not every month, or in only 1 or 2 months?
6. In the last 12 months, did you ever eat less than you felt you should because there wasn’t enough money for food? (Yes/No)
7. In the last 12 months, were you ever hungry, but didn’t eat, because there wasn’t enough money for food? (Yes/No)
8. In the last 12 months, did you lose weight because there wasn’t enough money for food? (Yes/No)
9. In the last 12 months did you or other adults in your household ever not eat for a whole day because there wasn’t enough money for food? (Yes/No)
10. (If yes to question 9) How often did this happen—almost every month, some months but not every month, or in only 1 or 2 months?
